# Thromboelastography for the evaluation of coagulation function in patients with hepatic echinococcosis during the perioperative period: a retrospective study

**DOI:** 10.3389/fonc.2025.1603148

**Published:** 2025-08-21

**Authors:** Xing Zheng, Li Yuan, Bingxian Wang, Genkui Li, Jing Wang, Jide A, Zongzhao He

**Affiliations:** ^1^ Department of Intensive Care Unit, Qinghai Provincial People’s Hospital, Xining, China; ^2^ Department of Anesthesiology, Qinghai Provincial People’s Hospital, Xining, China; ^3^ Department of Radiology, Hospital of Honghe State Affiliated to Kunming Medical University, Kunming, China; ^4^ Department of Medical, Qinghai Provincial People’s Hospital, Xining, China; ^5^ Department of General Surgery, Qinghai Provincial People’s Hospital, Xining, Qinghai, China

**Keywords:** coagulation abnormalities, hepatic echinococcosis, hypohepatia, platelet aggregation function, thromboelastography (TEG)

## Abstract

**Objective:**

To study the application of thrombolysis diagram (TEG) and routine coagulation test in the evaluation of coagulation function in patients with hepatic hydatid.

**Methods:**

The observation group consisted of 69 cases of hydatid liver patients undergoing elective combined segmenectomy, and the control group consisted of 69 healthy subjects. The correlation analysis of TEG, six coagulation items and PLT in the preoperative observation group and control group was conducted. The differences of TEG, hemagglutination six and PLT between the two groups were compared. TEG and coagulation function indexes of observation group were compared at different time during perioperative period.

**Results:**

In the control group, The time of blood cell aggregation (K) was negatively correlated with prothrombin time (PT), fibrinogen (FIB) and platelet count(PLT); The maximum strength of blood clot (MA) was positively correlated with PT, FIB and PLT; The rate of blood cell aggregation (Angle) is positively correlated with PT, FIB and PLT; the comprehensive index of blood coagulation (CI) was positively correlated with FIB and PLT. The reaction time of blood coagulation (R) of observation group was positively correlated with PT; MA was positively correlated with PT, FIB and PLT; MA was negatively correlated with thrombin time (TT). activated partial thromboplastin time (APTT), D-dimer (DD), PLT, R value, K value, Angle, MA and CI were compared between the two groups, and the differences were statistically significant (P<0.05). Compared with T1, APTT, R value, Angle and MA in observation group were significantly decreased at T2 and T3 (P<0.05); Compared with T1, PT, TT, FIB and K values had no significant changes (P>0.05).

**Conclusion:**

The coagulation function of liver hydatid patients was relatively low during the perioperative period, TEG is more sensitive to changes in perioperative coagulation function and can comprehensively understand the patient’s coagulation state.

## 1 Introduction

Hepatic echinococcosis is a prevalent zoonotic parasitic disease frequently observed in pastoral regions, such as Qinghai, Tibet, and Xinjiang, within China. Echinococcus has the ability to invade multiple organs, with the liver being the most commonly affected site, comprising approximately 75% of all documented cases (1). The liver plays a critical role in the synthesis of various coagulation factors and proteins within the body. In the event of liver disease, such conditions may lead to significant alterations in the patient’s coagulation system (2). Among the currently identified clotting factors, all except factor IV are synthesized through hepatic involvement. Moreover, the liver is involved in both the synthesis and inactivation of fibrinolytic and antifibrinolytic substances, thereby exerting a critical influence on the hemostatic and coagulation mechanisms within the body (3). As hepatic echinococcosis progresses, the lesion may compress or infiltrate the liver parenchyma, thereby leading to impaired liver synthetic function, as indicated by decreased levels of coagulation factors II, V, VII, and X, prolonged prothrombin time (PT) and activated partial thromboplastin time (APTT), and consequently elevated risk of intraoperative bleeding. Chronic inflammatory states are capable of activating the coagulation system, as indicated by elevated fibrinogen levels, and consequently enhance the risk of hypercoagulability. The intraoperative release of hydatid cyst fluid, which contains antigenic substances, may potentially lead to hyperfibrinolysis or disseminated intravascular coagulation (4). Conventional coagulation function tests necessitate laboratory processing, are time-intensive, neglect the dynamic interplay among the various components of the coagulation system, and are incapable of providing a holistic assessment of the entire coagulation process. The test results solely signify the precise time points at which coagulation begins or fibrin formation takes place, and are incapable of capturing the dynamic fluctuations in the rate and intensity of coagulation, as well as the fibrinolysis process. It is not feasible to quantitatively evaluate the balance between thrombosis and bleeding risks, nor can it adequately reflect the influence of cellular components, such as platelets, red blood cells, and white blood cells, on the coagulation process (5, 6). Thromboelastography (TEG) facilitates the dynamic observation of the entire process, including coagulation factor activation, fibrinogen network formation, platelet aggregation, blood clot strengthening, and blood clot dissolution, and offers a comprehensive and dynamic assessment of the coagulation functional status within the patient’s body (7). Currently, there is a limited number of clinical studies utilizing TEG in patients with hepatic echinococcosis. In this study, the observation group underwent thromboelastography (TEG) and testing of six coagulation parameters during the perioperative period. These indicators were then compared to assess changes in coagulation function among patients with hepatic echinococcosis throughout the perioperative phase. Conduct a comprehensive assessment of platelet function, fibrinogen levels, and fibrinolytic activity preoperatively to predict the risk of bleeding; continuously monitor the increase in the MA value for the identification of a latent hypercoagulable state. Intraoperative real-time monitoring facilitates the timely detection of hypofibrinogenemia, abnormal platelet function, and the early recognition of hyperfibrinolysis, thereby enabling more precise guidance for component-specific blood transfusion. The continuous postoperative increase in the MA value indicates the need for anticoagulation prophylaxis. Below is a detailed report on the application of TEG in evaluating the coagulation function of patients with hepatic echinococcosis during the perioperative period.

## 2 Data and methods

### 2.1 General information

A total of 69 patients with hepatic echinococcosis who were admitted to Qinghai Provincial People’s Hospital between July 2019 and October 2021 constituted the observation group, whereas 69 healthy individuals undergoing routine health examinations during the same period formed the control group. Inclusion criteria: The observation group comprised adult patients diagnosed with hepatic echinococcosis. Control group: Adult healthy individuals were randomly selected from those who underwent routine physical examinations. All participants met the health eligibility criteria and exhibited no pathological conditions in the cardiovascular system, liver, kidneys, brain, or other organs. All participants successfully completed the physical examination and had no documented history of cardiovascular, hepatic, renal, cerebral, or other organ-related diseases. Pregnant women were excluded from the statistical analysis. Criteria for exclusion:Co-occurrence of other systemic diseases. Participants who have received anticoagulant medications within one month prior to the study or are currently receiving anticoagulant treatment. Individuals diagnosed with hematological disorders. Chronic kidney diseases. Cardiovascular and cerebrovascular disorders. Individuals with known congenital blood coagulation disorders are included. Patients with systemic infections or incomplete TEG parameters and conventional coagulation function test indicators were excluded from the study.

### 2.2 Methods

#### 2.2.1 Standard coagulation function assays

The reagent kits for measuring activated partial thromboplastin time (APTT), prothrombin time (PT), thrombin time (TT), fibrinogen (FIB), D-dimer (DD), and fibrinogen degradation products (FDP), along with the internal quality control materials, calibrators, and the STA-R Evolution fully automatic coagulation analyzer, were all sourced from Stago Company, France. The six coagulation parameters of the patients were assessed at three specific perioperative time points: T1 (preoperatively), T2 (at the final release of hepatic portal occlusion), and T3 (immediately postoperatively).

#### 2.2.2 Thromboelastographic monitoring

The reaction time of blood coagulation (R), the time of blood cell aggregation (K), the rate of blood cell aggregation (Angle), the maximum strength of blood clot (MA), and the comprehensive index of blood coagulation (CI) were quantitatively assessed using the TEG5000 thromboelastography system, comprising its dedicated analyzer, reagents, and software, which were supplied by Hemoscope Corporation (United States).Thromboelastography (TEG) analyses were performed at time points T1 to T3.

#### 2.2.3 Statistical method

This analysis utilized a range of statistical methods to conduct hypothesis testing. For categorical variables (e.g., gender, ASA classification, and Child classification), the Pearson chi-square test or Fisher’s exact test was employed. The Kolmogorov-Smirnov test and the Shapiro-Wilk test were utilized to assess normality. For continuous variables, Based on the data distribution and variance homogeneity, The Welch t-test (for approximately normal distributions) or the Wilcoxon rank-sum test (for non-normal distributions) was applied. Specifically, in the comparison of general information across groups, A combination of Pearson’s chi-square test, Welch’s t-test, the Wilcoxon rank-sum test, and Fisher’s exact test was comprehensively utilized. For the comparison of liver function and thromboelastography (TEG) parameters, the Wilcoxon rank-sum test was consistently applied. For the comparison of the six coagulation parameters, a combination of the Welch t-test and the Wilcoxon rank-sum test was utilized. Count data were analyzed using the chi-square (χ²) test, and measurement data with skewed distributions were presented as median (interquartile range) [M (Q)].For correlation analysis, Pearson or Spearman correlation coefficients were computed, depending on the data type. Significance levels were indicated by asterisks (**** *p* < 0.0001, *** *p* < 0.001, ** *p* < 0.01, * *p* < 0.05).The significance threshold for all statistical tests was defined as α = 0.05.

## 3 Results

### 3.1 Comparison of baseline characteristics between the two patient groups

In the table comparing differences in general information (**Table 1**), No statistically significant differences were observed between the cystic echinococcosis (N = 41) and the alveolar echinococcosis (N = 28) with respect to gender (χ² = 2.196, *p* = 0.138), age (t = 0.384, *p* = 0.702), height (W = –1.111, *p* = 0.267), weight (t = 0.321, *p* = 0.749), ASA classification (*p* = 0.301), and Child–Pugh classification (*p* = 0.504); all *p* > 0.05.

**Table 1:** Comparison of general characteristics between the two patient groups.

Cystic echinococcosis (CE) N = 41^1^		Alveolar echinococcosis (AE) N = 28^1^	Statistical magnitude^2^	*P*-values ^2^
Gender			2.196	0.138
male	16 (39.02%)	16 (57.14%)		
female	25 (60.98%)	12 (42.86%)		
Age	40.32 ± 15.59	38.93 ± 14.14	0.384	0.702
Height	1.64 (1.58, 1.70)	1.65 (1.60, 1.70)	-1.111	0.267
Weight	61.40 ± 12.53	60.46 ± 11.32	0.321	0.749
ASA classification				0.301
II	9 (21.95%)	2 (7.14%)		
III	29 (70.73%)	24 (85.71%)		
IV	3 (7.32%)	2 (7.14%)		
Child-Pugh classification				0.504
A	40 (97.56%)	26 (92.86%)		
B	1 (2.44%)	1 (3.57%)		
C	0 (0.00%)	1 (3.57%)		

^1^n (%); Mean ± SD; Median (Q1, Q3).

^2^Pearson’s Chi-squared test; Welch Two Sample t-test; Wilcoxon rank sum test; Fisher’s exact test.

ASA classification: Preoperative Risk Assessment System for Patients. Child-Pugh classification: Grading Standards for Assessing Liver Reserve Function.

In the Comparative Table of Liver Function Differences (**Table 2**), No statistically significant differences were observed in alanine aminotransferase (ALT) (W = 0.049, *p* = 0.961), aspartate aminotransferase (AST) (W = –0.129, *p* = 0.898), or total bilirubin (TBIL) (W = –1.084, *p* = 0.278) between the cystic echinococcosis (N = 41) and the alveolar echinococcosis (N = 28), all *p* > 0.05.

**Table 2:** Comparison of liver function differences across different types.

Cystic echinococcosis (CE) N = 41^1^		Alveolar echinococcosis (AE) N = 28^1^	Statistical magnitude^2^	*P*-values ^2^
ALT	24.00 (18.00, 34.00)	20.50 (15.50, 67.50)	0.049	0.961
AST	23.00 (21.00, 26.00)	22.50 (19.00, 49.50)	-0.129	0.898
TBIL	10.60 (8.55, 17.35)	11.95 (9.70, 17.60)	-1.084	0.278

^1^Median (Q1, Q3).

^2^Wilcoxon rank sum test.

ALT, alanine aminotransferase; AST, aspartate aminotransferase; TBIL, total bilirubin.

### 3.2 Correlation analysis of routine coagulation function and thromboelastography in cystic echinococcosis and alveolar echinococcosis

In the Comparative Table of the Six Coagulation Parameters (**Table 3**), A statistically significant difference in international normalized ratio(INR) was observed between the cystic echinococcosis group (mean ± standard deviation: 1.01 ± 0.08) and the alveolar echinococcosis group (mean ± standard deviation: 1.06 ± 0.08) (t = –2.436, *p* = 0.018).A statistically significant difference in fibrinogen (FIB) was observed between the cystic echinococcosis group (median [Q1, Q3]: 2.42 [2.11, 2.97]) and the alveolar echinococcosis group (median [Q1, Q3]: 3.04 [2.68, 3.66]) (W = –3.043, *p* = 0.002).Other indicators, including prothrombin time (PT:t = 1.785, *p* = 0.080), activated partial thromboplastin time (APTT:t = –1.860, *p* = 0.070), thrombin time (TT:W = 0.893, *p* = 0.372), D-dimer (D-Di:W = 0.251, *p* = 0.802), and fibrinogen degradation products (FDP:W = –1.442, *p* = 0.149), did not show statistically significant differences (all *p* > 0.05).

**Table 3:** Correlation analysis of routine coagulation function between cystic echinococcosis and alveolar echinococcosis.

Cystic echinococcosis (CE) N = 41^1^		Alveolar echinococcosis (AE) N = 28^1^	Statistical magnitude^2^	*P*-values ^2^
PT	93.42 ± 13.73	86.83 ± 15.92	1.785	0.080
INR	1.01 ± 0.08	1.06 ± 0.08	-2.436	0.018
APTT	28.81 ± 3.65	31.18 ± 6.01	-1.860	0.070
FIB	2.42 (2.11, 2.97)	3.04 (2.68, 3.66)	-3.043	0.002
TT	18.50 (18.00, 19.10)	18.30 (17.85, 18.70)	0.893	0.372
D-Di	0.81 (0.60, 1.30)	0.76 (0.58, 1.36)	0.251	0.802
FDP	2.13 (1.54, 3.04)	2.45 (1.86, 4.46)	-1.442	0.149

^1^Mean ± SD; Median (Q1, Q3).

^2^Welch Two Sample t-test; Wilcoxon rank sum test.

PT, prothrombin time; INR, international normalized ratio; APTT, activated partial thromboplastin time; FIB, fibrinogen; TT, thrombin time; D-Di, D-dimer; FDP, fibrinogen degradation products.

In the Thromboelastography(TEG) Comparative Table (**Table 4**), No statistically significant differences were observed in the Reaction Time(R) (W = –0.581, *p* = 0.562), Clot Formation Time(K) (W = 0.722, *p* = 0.470), Alpha Angle(Angle) (W = –0.110, *p* = 0.912), Maximum Amplitude(MA) (W = –1.882, *p* = 0.060), and Coagulation Index(CI) (W = –0.214, *p* = 0.831) parameters between the cystic echinococcosis (N = 41) and the alveolar echinococcosis (N = 28), (all *p* > 0.05).

**Table 4:** Correlation analysis of thromboelastography between cystic echinococcosis and the alveolar echinococcosis.

Cystic echinococcosis (CE) N = 41^1^		Alveolar echinococcosis (AE) N = 28^1^	Statistical magnitude^2^	*P*-values ^2^
R	6.90 (5.50, 9.70)	7.50 (6.30, 9.85)	-0.581	0.562
K	2.00 (1.80, 2.90)	1.90 (1.40, 2.70)	0.722	0.470
Angle	62.80 (52.20, 66.70)	62.60 (50.60, 69.05)	-0.110	0.912
MA	61.40 (58.00, 64.60)	64.50 (59.20, 68.05)	-1.882	0.060
CI	-1.00 (-4.10, 0.40)	-0.60 (-3.40, 0.95)	-0.214	0.831

^1^Median (Q1, Q3).

^2^Wilcoxon rank sum test.

R, Reaction Time; K, Clot Formation Time; Angle, Alpha Angle; MA, Maximum Amplitude; CI, Coagulation Index.

### 3.3 Correlation Analysis between lesion size and conventional coagulation function as well as thromboelastography

**Figure 1:**
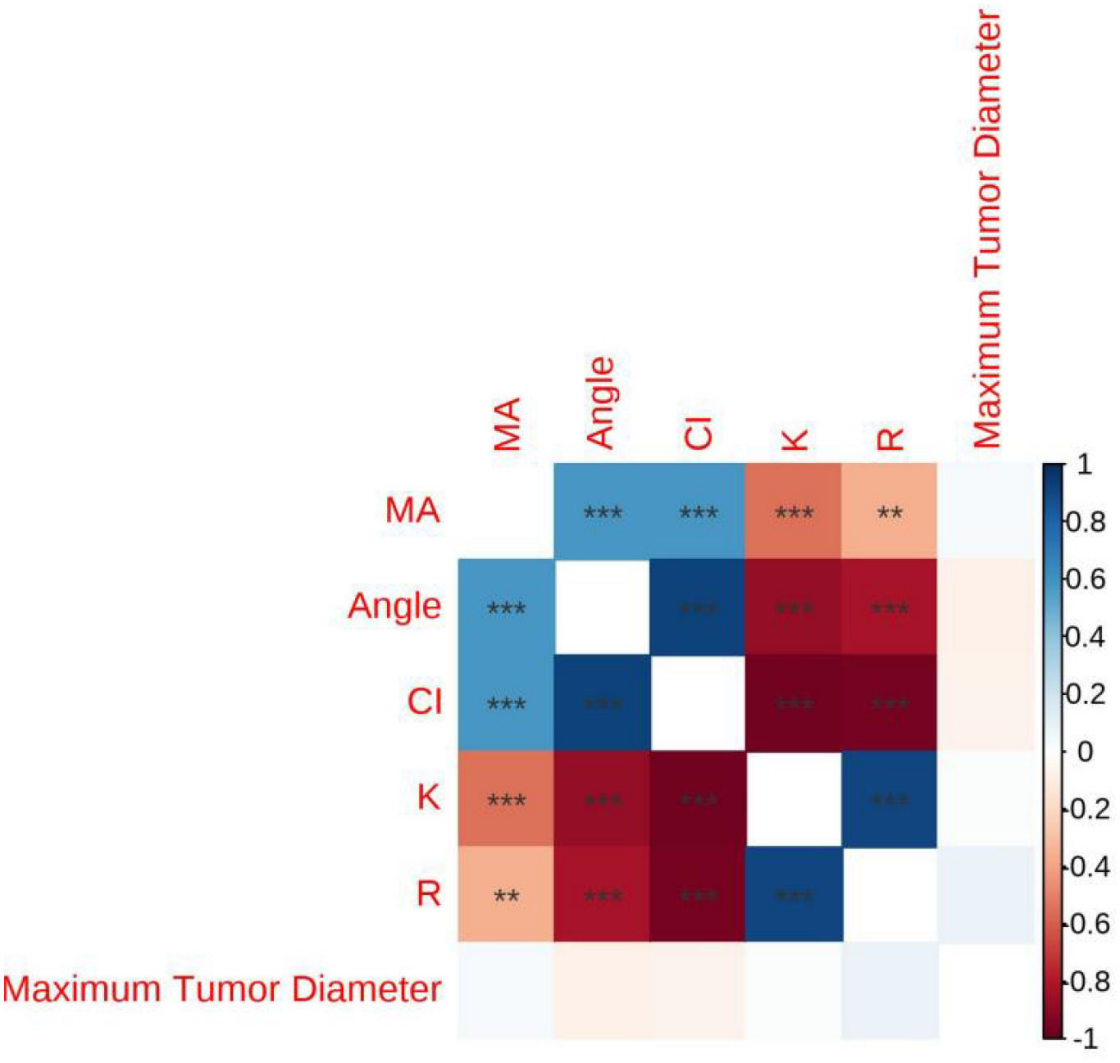
Heatmap of the correlation coefficient between lesion size and Thromboelastography (TEG).

In the Table of Correlation Between Lesion Size and Thromboelastography (TEG) (**Table 5**),The correlation coefficients between the maximum diameter of the lesion and R (r = 0.087), K (r = 0.019), Angle (r = –0.073), MA (r = 0.035), and CI (r = –0.064) were not statistically significant, as indicated by the absence of asterisks (all *p* > 0.05) (see **Figure 1**).

**Table 5:** Correlation analysis between lesion size and thromboelastography.

Thromboelastography (TEG)	R	K	Angle	MA	CI	Maximum Tumor Diameter
R						
K	0.91****					
Angle	-0.82****	-0.88****				
MA	-0.35**	-0.55****	0.59****			
CI	-0.96****	-0.96****	0.92****	0.59****		
Maximum Tumor Diameter	0.087	0.019	-0.073	0.035	-0.064	

R, Reaction Time; K, Clot Formation Time; Angle, Alpha Angle; MA, Maximum Amplitude; CI, Coagulation Index. "**" and "****" indicate the degree of statistical significance.

**Figure 2:**
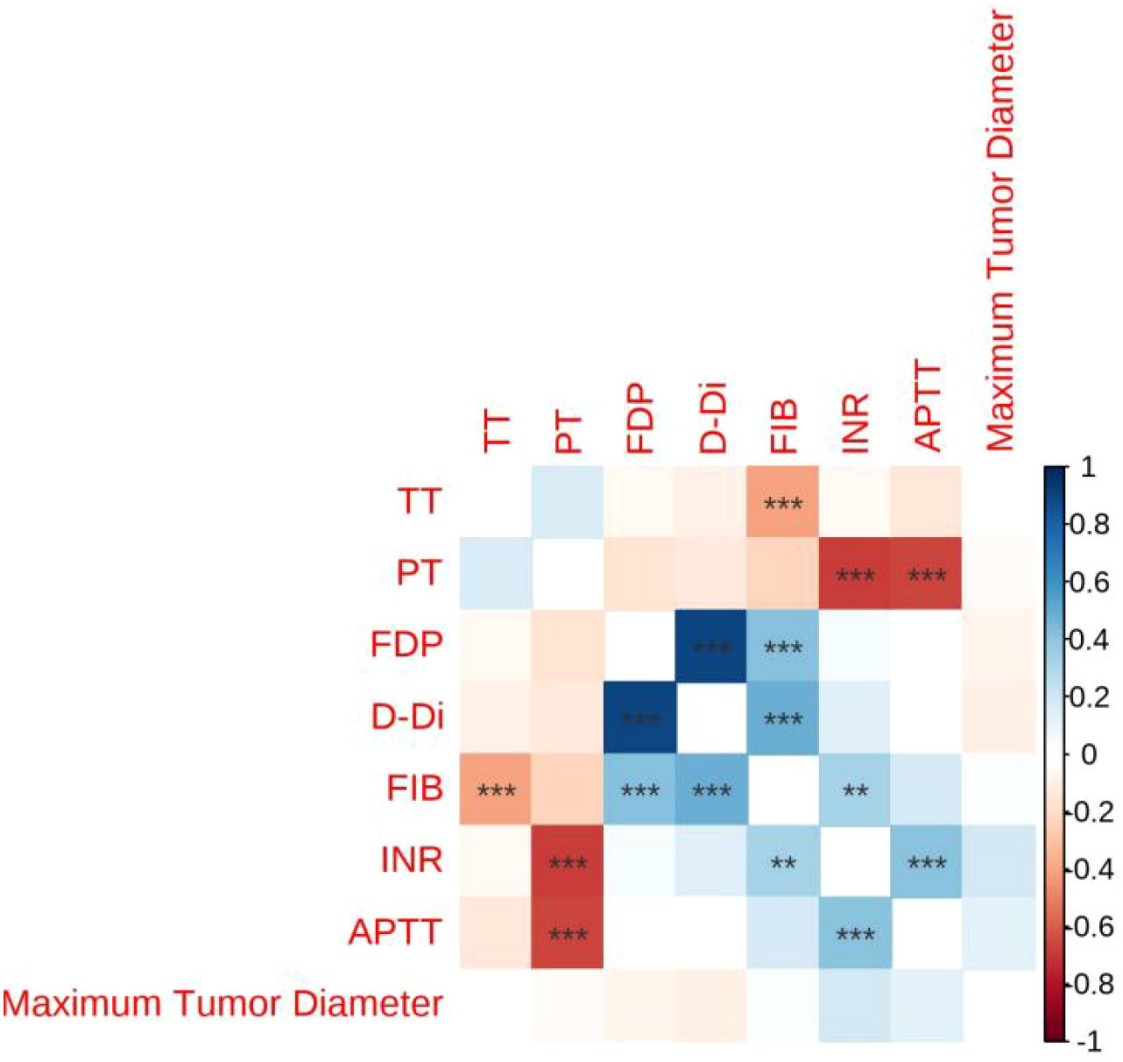
Heatmap showing the correlation coefficient between lesion size and coagulation function.

In the Table of Correlation Between Lesion Size and Coagulation Function (**Table 6**), The correlation coefficients between the maximum diameter of the lesion and PT (r = –0.044), INR (r = 0.19), APTT (r = 0.14), FIB (r = 0.035), TT (r = 0.0023), D-Di (r = –0.1), and FDP (r = –0.071) were not statistically significant, as indicated by the absence of asterisks (all *p* > 0.05) (see **Figure 2**).

**Table 6:** Correlation analysis between lesion size and routine coagulation function.

PT		inr	APTT	FIB	TT	D-Di	FDP	Maximum Tumor Diameter
PT								
INR	-0.69****							
APTT	-0.66****	0.41***						
FIB	-0.23	0.33**	0.19					
TT	0.18	-0.054	-0.13	-0.4***				
D-Di	-0.13	0.14	-0.0026	0.5****	-0.099			
FDP	-0.17	0.053	0.019	0.43***	-0.058	0.91****		
Maximum Tumor Diameter	-0.044	0.19	0.14	0.035	0.0023	-0.1	-0.071	

PT, prothrombin time; INR, international normalized ratio; APTT, activated partial thromboplastin time; FIB, fibrinogen; TT, thrombin time; D-Di, D-dimer; FDP, fibrinogen degradation products.

"**", "***" and "****"indicate the degree of statistical significance.

### 3.4 Correlation analysis of thromboelastography with conventional coagulation function and platelet count in the control group

The K value in the control group showed a negative correlation with platelet count (PLT), prothrombin time (PT), and fibrinogen (FIB).Alpha Angle(Angle) showed a positive correlation with PLT, PT, FIB.Maximum Amplitude(MA) is positively correlated with PLT, PT and FIB.(*P*<0. 05) (**Table 7**).

**Table 7:** Correlation analysis of TEG parameters, conventional coagulation function, and platelet count in the control group.

Thromboelastography (TEG)	APTT (s)	PT(s)	TT(s)	FIB(g)	PLT(×10^9^/L)
	*r P*	*r P*	*r P*	*r P*	*r P*
MA(mm)	0.054 0.662	0.341 0.004	0.045 0.715	0.560 0.000	0.296 0.013
R(min)	-0.049 0.687	0.055 0.645	0.037 0.762	0.067 0.585	-0.153 0.208
K(min)	-0.002 0.986	-0.270 0.025	0.028 0.818	-0.462 0.000	-0.316 0.008
Angle(deg)	0.011 0.927	0.286 0.017	0. 062 0.614	0.466 0.000	0. 319 0.008
CI	0.049 0.691	0.226 0.062	-0.018 0.883	0.364 0.002	0.262 0.030

R, Reaction Time; K, Clot Formation Time; Angle, Alpha Angle; MA, Maximum Amplitude; CI, Coagulation Index.

### 3.5 Correlation analysis of thromboelastography, conventional coagulation function, and platelet count in the observation group

The R value in the observation group showed a positive correlation with prothrombin time (PT).Maximum Amplitude(MA) showed a positive correlation with prothrombin time (PT), fibrinogen (FIB), and platelet count (PLT).MA is negatively correlated with thrombin time(TT).(*P*<0. 05) (**Table 8**).

**Table 8:** Correlation analysis of thromboelastography, conventional coagulation function, and platelet count in the observation group.

Routine Coagulation Function	MA(mm)		R (s)	
	*r*	*P*	*r*	*P*
APTT(s)	0.174	0.154	0.093	0.446
PT(s)	0.286	0.017	0.243	0.045
TT(s)	-0.336	0.005	-0.067	0.585
FIB(g)	0.580	0.000	0.101	0.407
PLT(×10^9^/L)	0.427	0.000	-0.058	0.633

APTT, activated partial thromboplastin time; PT, prothrombin time; TT, thrombin time; FIB, fibrinogen; PLT, platelet count.

### 3.6 Comparison of coagulation parameters, platelet count, and thromboelastography between the two patient groups

The activated partial thromboplastin time (APTT), R value, and K value in the observation group were significantly prolonged compared to those in the control group. The platelet count (PLT) was significantly lower compared to that of the control group. Thrombin time (TT), D-dimer (D-DI), Alpha Angle(Angle), Maximum Amplitude(MA) and coagulation index (CI) were significantly shorter compared to those in the control group.(*P*<0. 05) (**Table 9**).

**Table 9:** Comparison of coagulation parameters, platelet count, and thromboelastography between the two patient groups [median (IQR)].

	Control Group (*n*=69)	Observation Group (*n*=69)	*P*值
ATPP (s)	26.1 (24.4∼28.7)	29.6 (25.9∼32.0)^a^	0.000
PT (s)	12.0 (11.4∼12.8)	11.8 (11.4∼12.2)	0.116
TT (s)	19.1 (18.1∼20.1)	18.3 (17.9∼19.1)^a^	0.015
FIB (g/L)	2.8 (2.4∼3.6)	2.7 (2.2∼3.6)	0.370
D-DI (mg/L)	1.2 (0.9∼2.2)	0.8 (0.6∼1.3)^a^	0.000
FDP (g/L)	2.5 (1.6∼4.1)	2.1 (1.7∼3.8)	0.831
PLT (×10^9^/L)	253 (209∼294)	206 (155∼257)^a^	0.001
R值 (min)	5.4 (4.2∼6.5)	7.3 (5.7∼9.7)^a^	0.000
K值 (min)	1.5 (1.2∼1.8)	2.0 (1.6∼2.8)^a^	0.000
Angle角 (deg)	67.9 (63.7∼72.0)	62.8 (52.0∼66.8)^a^	0.000
MA值 (mm)	70.4 (65.3∼73.5)	62.7 (58.0∼66.0)^a^	0.000
CI	1.9 (0.5∼3.0)	-0.9 (-3.8∼0.5)^a^	0.000

APTT, activated partial thromboplastin time; PT, prothrombin time; TT, thrombin time; FIB, fibrinogen; D-Di, D-Dimer Level; FDP, Fibrin Degradation Products; PLT, platelet count; R, Reaction Time; K, Clot Formation Time; Angle, Alpha Angle; MA, Maximum Amplitude; CI, Coagulation Index.

The superscript letter "a" in dicates that the observation group and the control group were compared, and there were significant contrasting changes, with statistically significant differences.

### 3.7 Changes in coagulation parameters at different time points during the perioperative period in the observation group

Changes in Coagulation Parameters at Different Time Points During the Perioperative Period in the Observation Group, Compared with T1, activated partial thromboplastin time (APTT), R value, Angle, and MA were significantly reduced at T2 and T3 (P < 0.05).Compared with T1, no significant changes were observed in prothrombin time (PT), thrombin time (TT), fibrinogen (FIB), or Clot Formation Time(K) (*P* > 0.05) (**Table 10**).

**Table 10:** Changes in coagulation parameters at different time points during the perioperative period in the observation group[M (IQR)].

Project Initiative	T1	T2	T3
APTT (S)	29.6 (25.9∼32.0)	28.1 (27.0∼30.1)	27.6 (24.6∼30.5)
PT (S)	11.8 (11.4∼12.2)	12.2 (11.0∼13.6)	10.8 (10.3∼11.5)
TT (S)	18.3 (17.9∼19.1)	18.6 (17.2∼19.4)	17.6 (16.7∼18.1)
FIB (g/L)	2.7 (2.2∼3.6)	2.6 (2.1∼3.5)	2.8 (2.5∼3.2)
R (min)	7.3 (5.7∼9.7)	6.1 (4.6∼8.5)^a^	5.9 (1.3∼12.4)^a^
K (min)	2.0 (1.6∼2.8)	1.9 (1.6∼2.3)	1.8 (1.5∼2.3)
Angle (deg)	62.8 (52.0∼66.8)	61.5 (56.5∼65.6)^a^	60.7 (58.4∼63.2)^a^
MA (mm)	62.7 (58.0∼66.0)	61.2 (56.3∼65.1)^a^	60.1 (55.8∼63.9)^a^

APTT, activated partial thromboplastin time; PT, prothrombin time; TT, thrombin time; FIB, fibrinogen; R, Reaction Time; K, Clot Formation Time; Angle, Alpha Angle; MA, Maximum Amplitude.

## 4 Discussion

Radical hepatectomy is widely recognized as the preferred and most effective surgical approach for the treatment of hepatic echinococcosis. It not only enables complete eradication of the lesion, but also contributes to prolonging the patient’s lifespan and enhancing their quality of life. The most prevalent mode of invasion for echinococcosis lesions is intrahepatic metastasis. Erosion of the perilesional hepatic tissue and extensive damage to the liver parenchyma. The synthetic and secretory functions of hepatocytes are markedly impaired, characterized by prothrombin deficiency and disturbances in the synthesis of coagulation factors. Ultimately, this resulted in a pronounced bleeding tendency in the patient. This is not conducive to the successful and efficient progression of the surgical procedure (8).Routine coagulation function tests are capable of reflecting only the initial phase of the coagulation process. It is unable to provide information regarding platelet function, thrombus strength, or fibrinolytic activity. However, the advantage of TEG lies in its utilization of whole blood for analysis, thereby accounting for the combined effects of anticoagulants, coagulation factors, and platelets. It offers distinct advantages in assessing coagulation function and guiding blood transfusion therapy, among other clinical applications (9–12).This article evaluates the coagulation function of patients with hepatic echinococcosis through the combined application of conventional coagulation assays and thromboelastography (TEG).

The results of this study demonstrate that there is no statistically significant difference in the comparison of general data. Among the collected cases, the Child-Pugh classification for polycystic liver disease and vesicular liver was uniformly classified as Class A. No statistically significant differences were observed in liver function, conventional coagulation parameters, or thromboelastography (TEG) between the two groups. Correlation analysis between conventional coagulation parameters and thromboelastography (TEG) demonstrates. A strong correlation exists between thromboelastography (TEG) and conventional coagulation parameters in healthy individuals undergoing routine physical examinations. The Clot Formation Time (K) demonstrates a negative correlation with platelet count (PLT), prothrombin time (PT), and fibrinogen (FIB).Alpha Angle demonstrates a positive correlation with platelet count (PLT), prothrombin time (PT), and fibrinogen (FIB).Maximum Amplitude (MA) is positively correlated with platelet count (PLT), prothrombin time (PT) and fibrinogen (FIB).Coagulation Index (CI) demonstrates a positive correlation with platelet count (PLT) and fibrinogen (FIB).The Reaction Time (R) in the observation group demonstrated a positive correlation with prothrombin time (PT).Maximum Amplitude (MA) demonstrates a positive correlation with prothrombin time (PT), fibrinogen (FIB), and platelet count (PLT).Maximum Amplitude (MA) is negatively correlated with thrombin time (TT).A strong correlation exists between MA and conventional coagulation parameters in patients with hepatic echinococcosis, whereas the correlations of other indicators are not statistically significant. Therefore, for patients with hepatic echinococcosis, preoperative assessment of coagulation function through the integration of routine coagulation tests and thromboelastography (TEG) can provide a more comprehensive evaluation of the patient’s hemostatic status. Through comparison of thromboelastography (TEG) and conventional coagulation parameters between the two groups, it was observed that, In patients with hepatic echinococcosis, activated partial thromboplastin time (APTT), R value, and K value are prolonged, platelet count (PLT) is reduced, while Angle, maximum amplitude (MA), and coagulation index (CI) are shortened. The coagulation function is in a relatively hypocoagulable state. Elevated activated partial thromboplastin time (APTT) in patients with hepatic echinococcosis, Reflects abnormal activity and levels of coagulation factors in the extrinsic coagulation pathway. It serves as an important indicator of the severity of hepatic lesions (13–15).In addition to causing a deficiency of coagulation factors, hepatic echinococcosis is also associated with decreased platelet count and impaired platelet function. The research findings indicate that maximum amplitude (MA) is significantly reduced in patients with hepatic echinococcosis. Platelets are the primary components that become activated early and initiate subsequent cascade reactions during the hemostasis and coagulation process. The decrease in platelet count among patients with hepatic echinococcosis is primarily attributed to liver cirrhosis, portal hypertension, and splenomegaly. The sequestration of platelets within the spleen and the enhanced activity of splenic macrophages contribute to increased platelet destruction in the splenic sinusoids (16–19).The perioperative coagulation function tests conducted on patients with hepatic echinococcosis in this study demonstrated that, Compared with T1, APTT, R value, Angle, and MA were significantly reduced at T2 and T3.Patients with hepatic echinococcosis exhibit varying degrees of coagulation dysfunction during the perioperative period. The patient’s coagulation profile should be optimized prior to surgery. Address and correct the existing coagulation abnormalities. To minimize intraoperative blood loss and reduce the requirement for blood product transfusions (20, 21).Due to multiple contributing factors—including prolonged anesthesia duration, surgical trauma, intraoperative bleeding, large-volume and rapid fluid administration, and intraoperative hypothermia—patients are at increased risk of developing coagulation dysfunction both during and following surgery. Especially when the lesion resection is extensive, and the hemodynamic alterations resulting from hepatic blood flow occlusion contribute to more pronounced coagulation abnormalities (22, 23).

Although thromboelastography (TEG) demonstrates clinical utility in perioperative coagulation management for patients with hepatic echinococcosis. However, due to the unique nature of liver diseases, the impact of high-altitude conditions, and various surgical influences, its application presents the following limitations and shortcomings. The MA (maximum amplitude) value in thromboelastography (TEG) primarily reflects platelet function and fibrinogen concentration. However, in patients with liver disease, hypofibrinogenemia may be masked by compensatory platelet function (as indicated by normal or mildly reduced MA values), thereby concealing the actual risk (24, 25).Patients with hepatic echinococcosis frequently experience impaired vitamin K absorption as a result of bile stasis (manifested by prolonged prothrombin time [PT]).However, the prolonged R time (representing coagulation initiation time) in thromboelastography (TEG) is unable to differentiate whether this is attributable to factor VII deficiency, hepatic synthetic dysfunction, or disseminated intravascular coagulation (DIC) (26).Hypersplenism is frequently observed in the advanced stages of liver disease. However, the LY30 (fibrinolytic index) in thromboelastography (TEG) may appear deceptively normal due to interference from platelet microaggregates (27).Additionally, the hypoxic, cold, and hypobaric environmental conditions encountered in high-altitude regions can lead to various physiological and pathological alterations in the human body. Hypoxia stimulates erythropoietin (EPO) secretion, resulting in the development of secondary polycythemia (28).Low temperature promotes enhanced platelet aggregation, thereby increasing blood viscosity. Hemoconcentration results in elevated levels of coagulation factors (e.g., fibrinogen and factor VIII), which subsequently reduces fibrinolytic activity and predisposes to thrombosis. All of these factors may confound the interpretation of thromboelastography (TEG) results. Thromboelastography (TEG) lacks reference ranges specific to liver disease. Its clinical utility resides in the assessment of dynamic trends rather than in isolation-based diagnosis. Therefore, integrating multimodal monitoring with clinical evaluation is essential for optimizing coagulation management and minimizing associated risks.

The limitations of this study include its retrospective design, which focuses exclusively on coagulation and thereby presents inherent constraints. Furthermore, the sample size is relatively limited and warrants expansion in future research. In future studies, the sample size will be expanded, and a comprehensive evaluation of the severity of liver function impairment will be performed.

In conclusion, patients with hepatic echinococcosis exhibit a relatively hypocoagulable coagulation status, highlighting the necessity for close perioperative monitoring of coagulation function. Thromboelastography (TEG) enables comprehensive and dynamic evaluation of the entire coagulation and fibrinolysis process. Achieve the transition from “empirical blood transfusion” to “precision-based coagulation management” during surgical procedures. It is particularly indicated for patients with concomitant liver dysfunction, elevated intraoperative bleeding risk, or those in resource-constrained settings. It serves as a critical instrument for enhancing patient outcomes and reducing healthcare expenditures (29).Provide appropriate guidance for the accurate diagnosis, intervention, and treatment of patients with coagulation disorders.

## Data Availability

The original contributions presented in the study are included in the article/supplementary material. Further inquiries can be directed to the corresponding authors.
